# Descriptive Evaluation of Male Emergency Department Patients in the United States With Gonorrhea and Chlamydia

**DOI:** 10.7759/cureus.11244

**Published:** 2020-10-29

**Authors:** Justin M Elkins, Santiago Cantillo-Campos, Cheryl Thompson, Michael Mohseni, Johnathan M Sheele

**Affiliations:** 1 Emergency Medicine, Mayo Clinic, Jacksonville, USA; 2 Nutrition, Case Western Reserve University, Cleveland, USA

**Keywords:** chlamydia trachomatis, emergency department, male sex, neisseria gonorrhoeae, sexually transmitted infection, treatment, antibiotics, emergency medicine, sexually transmitted disease, diagnostic testing

## Abstract

Introduction

Sexually transmitted infections are commonly tested for in the emergency department (ED), but diagnostic test results are often unavailable during the clinical encounter.

Methods

We retrospectively reviewed health records of 3,132 men ≥18 years that had an emergency department visit in northeast Ohio between April 18, 2014 and March 7, 2017. All subjects underwent testing for Neisseria gonorrhoeae and Chlamydia trachomatis. Independent t-tests and chi-square analyses were performed as well as multivariable regression analysis.

Results

On univariable analysis, men with N gonorrhoeae and/or C trachomatis, compared with uninfected men, were younger (25.9 vs 32.4 years), more likely to be of Black race (91.7% vs 85.6%), less likely to be married (3.7% vs 10.2%), less likely to arrive to the ED by ambulance or police (1.7% vs 4.1%), and more likely to be diagnosed with a urinary tract infection (8.3% vs 3.7%), to be treated for gonorrhea and chlamydia in the ED (84.6% vs 54.9%), and to have higher emergency severity index (ESI) scores (3.8 vs 3.6) (P ≤ .03 for all). On urinalysis, men infected with N gonorrhoeae and/or C trachomatis had significantly more white blood cells (55.1 vs 20.9); more mucus (1.3 vs 1.2); higher leukocyte esterase (1.5 vs .4); fewer squamous epithelial cells (.6 vs 1.4); higher urobilinogen (1.1 vs .8); higher bilirubin (.09 vs .05); and more protein (.4 vs .3) (P ≤ .04).

Conclusions

Demographic and urinalysis findings can be associated with an increased odds of men being infected with N gonorrhoeae and/or C trachomatis.

## Introduction

*Neisseria gonorrhoeae* and *Chlamydia trachomatis *are among the most common notifiable infectious diseases in the US [1]. In 2018, the prevalence of *C trachomatis *reached one of the highest levels reported in US history, and the rates of gonorrhea have increased 63% since 2014 [1-3]. *N gonorrhoeae* and *C trachomatis* are increasing primarily among persons 25 years of age or younger and those of Black race [2]. Additional risk factors for *N gonorrhoeae* and *C trachomatis* in men include having more sexual partners, having sex with men, and being publicly insured [4,5]. In emergency departments (EDs), the incidence of gonorrhea is more prevalent and increasing among male versus female patients EDs [1].

Nucleic acid amplification tests (NAATs) are the recommended method for diagnosing *N gonorrhoeae *and *C trachomatis* [3]. However, most NAAT results are unavailable during the clinical encounter in the ED. Thus, ED clinicians must decide which patients require empiric antibiotic treatment for *N gonorrhoeae* and *C trachomatis* before the testing results are available. Although long-term complications of untreated sexually transmitted infections (STIs) in men are infrequent, they can include penile lymphangitis, penile edema, periurethral abscesses, epididymitis, disseminated gonococcal infection, prostatitis, and post-inflammatory urethral strictures [3,6]. Current recommendations are for all patients diagnosed or suspected of having *N gonorrhoeae* or *C trachomatis* to be treated if there is a high risk of failure to follow-up as an outpatient [3,7]. However, identifying those at risk for loss to follow-up is difficult [8, 9]. Thus, ED patients continue to be overtreated with antibiotics for both *N gonorrhoeae* and *C trachomatis* [7,10].

Men infected with *N gonorrhoeae* and *C trachomatis* can have inflammatory changes in the urine including higher levels of urine leukocyte esterase [11-15]. Most research involving *N gonorrhoeae* and *C trachomatis* in men come from ambulatory care centers or STI clinics with fewer epidemiologic investigations occurring in the ED patients. The objective of the study was to examine the demographic and clinical variables associated with men infected with *N gonorrhoeae* and *C trachomatis* in the ED.

## Materials and methods

Institutional review board approval was received by University Hospitals, and informed consent was waived for this retrospective study. The dataset included 75,000 ED patient encounters that occurred between April 18, 2014 and March 7, 2017. All patients in the data set were at least 18 years of age and either had a urinalysis and urine culture or were tested for gonorrhea, chlamydia, or trichomonas; however, we only analyzed men who received testing for *N gonorrhoeae* or *C trachomatis*. The dataset did not contain patients that were treated but not tested for STIs. Erroneous, incomplete, and missing results were not included in the analysis. An analysis from this dataset has previously been published [16].

Patients were considered infected or uninfected for *N gonorrhoeae* and *C trachomatis* on the basis of their Aptima Combo2 NAAT (Hologic, Inc, Marlborough, MA, USA) results. Patients were considered positive for T vaginalis if they had a positive NAAT result or if the parasite was observed on microscopy in the urine or a wet-mount preparation. Patients were considered negative for T vaginalis only if their NAAT results were negative. Patients were considered to have a urinary tract infection (UTI) if their records included an ED International Classification of Diseases (ICD), Ninth Revision or Tenth Revision diagnostic code: N30.90, O23.10, O86.22, N30.00, N30.91, N30, N30.0, N30.01, N30.90, N30.9, N39.0, O23.10, O23.40, O86.20, O08.83, O03.38, O03.88, O04.88, O86.2, O86.29, 595.0, 595.9, 595.89, 599.0, 639.8, 646.60, or 646.64). Triage data was not included in the dataset, but the reason for the ED visit at the initial patient point of contact was recorded. Text searches were performed for the following keywords recorded at the patient ED point of contact using Excel (Microsoft Corp, Redmond, WA, USA). Patients given ceftriaxone or cefixime plus azithromycin or an outpatient prescription for doxycycline were considered treated for *N gonorrhoeae* and *C trachomatis* in the ED. Patients given metronidazole either in the ED or as an outpatient prescription were also noted.

Due to laboratory variation in how urine red blood cells (RBCs) and white blood cells (WBCs) were reported, the dataset included either the actual number of cells counted or the mean number of cells if a range was reported. For patients with urine WBC and RBC with >100 cells/high-power field (HPF), the value was changed to 101 cells/HPF. The urinalysis included: bacteria (0-4+), bilirubin (0-3+), blood (0-3+), glucose (present or absent), ketones (0-3+ (with “trace” recorded as 0.5)), leukocyte esterase (0-3+), squamous epithelial cells/HPF, nitrite (positive or negative), protein (0-3+ (“trace” recoded as 0.5 for the analysis)), urobilinogen (<2 (recorded as 0), 2, 4, 8, and 12 mg/dL), and urine pH (5-9).

Data analysis

Continuous variables were summarized with mean and standard deviation (SD) and analyzed using the independent t-test. Categorical variables were summarized with frequencies and percentages and analyzed using the Fisher’s exact test. Stepwise regression analysis was performed using age, race (Black vs non-Black), type of health insurance, marital status (married/life partner, single, or widowed/separated/divorced), if the patient has a documented primary care physician, emergency severity index score (1-5), and how the patient got to the ED (private vehicle, public transportation, walked, or by police/emergency medical services [EMS]) to identify the variables for a multivariable regression model. The multivariable regression model included age (years), race, and emergency severity index (ESI). Analyses were performed using JMP Pro 14 software (SAS Institute Inc, Cary, NC, USA), and statistical significance was set at an α level of <.05.

## Results

Patient characteristics are summarized in Table 1. There were 3,132 men included in the analysis. A total of 14.6% (457/3,130) of the men tested positive for *C trachomatis*, 13.3% (418/3,121) tested positive for *N gonorrhoeae*, and 3.6% (114/3,132) were coinfected with *N gonorrhoeae* and *C trachomatis*. Overall, 24.3% (761/3,132) of men tested in the ED for *N gonorrhoeae* and/or *C trachomatis*, had positive results for one or both diseases. The mean age of men undergoing testing for *N gonorrhoeae* and *C trachomatis* was 31.0 years (10.9), the majority were of Black race, arrived to the ED by private vehicle, and were single.

**Table 1:** Patient demographics and triage characteristics ED: Emergency department; EMS: Emergency medical services; SD: Standard deviation; y: year.

Variable	Mean (SD) or % (N)
Age, mean (SD), y	31.0 (10.9) (n = 3,132)
Race	
Black	87.1% (2,709/3,112)
White	11.2% (349/3,112)
Asian	.2% (7/3,112)
Other	1.5% (47/3,112)
Method of ED arrival	
EMS/police	3.5% (109/3,104)
Public transportation	2.5% (78/3,104)
Car	91.3% (2,834/3,104)
Walked	2.7% (83/3,104)
Marital status	
Married	8.5% (267/3,131)
Life partner	.03% (1/3,131)
Single	87.7% (2,746/3,131)
Widowed	.1% (4/3,131)
Separated	1.0% (30/3,131)
Divorced	2.2% (68/3,131)
Hour of ED visit (0-23)	13.1 (6.2) (n = 3,132)

C trachomatis

Patients infected with *C trachomatis* were younger (25.2 vs 31.8 years), of Black (vs not Black) race (91.0% vs 86.4%), less likely to be married (2.9% vs 9.6%), had higher emergency severity index (ESI) scores (3.8 vs 3.6), and were more likely to be diagnosed with a UTI (7.9% vs 4.3%) than those without *C trachomatis* on univariable analyses (P ≤ .006 for all) (Table 2). Younger age and diagnosed with a UTI were the only significant variables on regression analyses between those with and without *C trachomatis* (P < .001).

**Table 2:** Demographic and clinical variables and their association with Chlamydia trachomatis in men dL: Deciliter; ED: Emergency department; EMS: Emergency medical services; HPF: High-power field; mg: milligram; NA: Not applicable; OR: Odds ratio; PCP: Primary care physician; SD: Standard deviation; STD: Sexually transmitted disease; STI: Sexually transmitted infection; UTI: Urinary tract infection; vs: versus; WBC: White blood cell; y: year. Adjusting for age, race, and emergency severity index score. ORs for continuous variables are stated as per unit change in regressor.

	+ C trachomatis	− C trachomatis	P Value	Adjusted OR (95% CI)	Adjusted P Value
Age, mean (SD), y	25.2 (6.4) (n = 457)	31.8 (11.2) (n = 2,673)	< .001	.91 (.90-.93)	< .001
Black race (vs not Black)	91.0% (414/455)	86.4% (2,293/2,655)	.006	1.40 (.99-1.99)	.06
Married/life partner (vs not married)	2.9% (13/454)	9.6% (255/2,660)	< .001	.67 (.37-1.22)	.19
Emergency severity index score, mean (SD), level 1-5	3.8 (.5) (n = 435)	3.6 (.6) (n = 2,524)	< .001	1.13 (.95-1.35)	.17
Having a (PCP) (vs none)	16.9% (77/457)	18.8% (503/2,673)	.33	.84 (.63-1.11)	.21
Arrived to ED by EMS/police (vs other)	2.0% (9/455)	3.8% (100/2,647)	.05	.56 (.26-1.19)	.13
ED triage pain scale, mean (SD) (0-10)	2.6 (3.6) (n = 24)	3.4 (3.8) (n = 234)	.31	1.02 (.77-1.35)	.89
Mentioned “STD,” “STI,” “sexually transmitted,” “penis,” or “discharge” at ED point of contact (vs not)	59.1% (270/457)	45.2% (1,208/2,673)	< .001	1.44 (1.15-1.81)	.002
Mentioned “UTI,” “bladder infection” or “pain,” “urinary tract infection,” “cystitis,” “dysuria,” “frequency” or “frequent urination,” or “urgency” at ED point of contact (vs not)	3.9% (18/457)	4.8% (127/2,673)	.55	.96 (.54-1.70)	.88
Mentioned “abdominal pain,” “abdominal discomfort,” or “abdominal cramping” at ED point of contact (vs not)	5.0% (23/457)	5.9% (158/2,673)	.52	1.02 (.63-1.65)	.94
Diagnosed in the ED with a urinary tract infection (vs not)	7.9% (36/457)	4.3% (114/2,673)	.002	2.68 (1.75-4.10)	< .001
Treated with metronidazole in the ED or given it as an outpatient prescription (vs not)	35.4% (162/457)	30.2% (808/2,673)	.03	1.12 (.90-1.41)	.31
Treated for gonorrhea and chlamydia (vs not)	81.2% (371/457)	58.8% (1,573/2,673)	< .001	2.67 (2.05-3.47)	< .001
Infected with N gonorrhoeae (vs not)	25.1% (114/455)	11.4% (304/2,664)	< .001	1.93 (1.49-2.51)	< .001
Infected with T vaginalis (vs not)	8.0% (9/112)	8.7% (59/677)	> .99	1.09 (.47-2.55)	.84
Urinalysis					
WBC count (cells/HPF), mean (SD)	42.1 (35.5) (n = 198)	29.9 (37.5) (n = 846)	< .001	1.01 (.99-1.05)	.33
WBC clumps present (vs not)	6.6% (13/197)	9.3% (78/837)	.26	.65 (.33-1.25)	.19
Mucus (0, 1+, 2+, 3+, or 4+), mean (SD)	1.1 (1.3) (n = 198)	1.2 (1.1) (n = 846)	.19	1.00 (.88-1.12)	.95
Leukocyte esterase (0, 1+, 2+, or 3+), mean (SD)	1.1 (1.0) (n = 246)	0.6 (1.0) (n = 1,593)	< .001	1.56 (1.38-1.77)	< .001
Bacteria (0, 1+, 2+, 3+, or 4+), mean (SD)	0.5 (.7) (n = 198)	0.6 (.9) (n = 845)	.02	.91 (.74-1.13)	.41
Nitrite positive (vs negative)	0.4% (1/250)	2.1% (33/1,606)	.08	.43 (.06-3.28)	.41
Blood (0, 1+, 2+, or 3+), mean (SD)	0.3 (.7) (n = 250)	0.3 (.7) (n = 1,587)	.92	1.17 (.96-1.42)	.11
Red blood cells (cells/HPF), mean (SD)	9.7 (21.8) (n = 198)	12.1 (24.7) (n = 843)	.18	1.00 (.99-1.01)	.63
Squamous epithelial cells (cells/HPF), mean (SD)	0.8 (3.3) (n = 57)	1.2 (2.5) (n = 220)	.39	.98 (.87-1.11)	.74
pH (5-9), mean (SD)	6.2 (.9) (n = 250)	6.0 (.9) (n = 1,607)	.004	1.10 (.95-1.28)	.20
Bilirubin (0, 1+, 2+, or 3+), mean (SD)	0.1 (.5) (n = 250)	0.04 (.3) (n = 1,602)	.06	1.51 (1.08-2.11)	.02
Urobilinogen (0, 2, 4, 8, or 12 mg/dL), mean (SD)	1.1 (1.7) (n = 250)	0.8 (1.6) (n = 1,606)	.01	1.04 (.96-1.13)	.34
Protein (0, .5+, 1+, 2+, 3+), mean (SD)	.4 (.6) (n = 250)	.3 (.6) (n = 1,606)	.18	1.23 (.98-1.54)	.07
Glucose present (vs not)	.4% (1/250)	5.2% (83/1,604)	< .001	.15 (.02-1.12)	.06
Ketones (0, .5+, 1+, 2+, 3+), mean (SD)	.1 (.4) (n = 250)	.1 (.4) (n = 1,604)	.79	.97 (.66-1.41)	.86

On univariable analysis, significant findings between those with and without *C trachomatis* in the urinalysis were higher WBCs, higher leukocyte esterase, higher urine pH, higher urobilinogen, fewer bacteria, and less likely to have glucose (P ≤ .02). On regression analysis only higher leukocyte esterase and urine bilirubin were significant (P ≤ .02). There were no significant differences between those with or without *C trachomatis* and infection with *T vaginalis*.

N gonorrhoeae

On univariable analysis, patients infected with *N gonorrhoeae* were younger (26.4 vs 31.5 years of age), of Black (vs not Black) race (93.0% vs 86.1%), less likely to be married (4.6% vs 9.3%), had higher ESI scores (3.8 vs 3.6), were less likely to come to the ED by emergency medical services (EMS) or police (1.5% vs 3.8%), and more likely to be diagnosed with a UTI (8.6% vs 4.2%) than those without *N gonorrhoeae* (P ≤ .01 for all) (Table 3). Younger age, Black race, higher ESI, and diagnosed with a UTI were significant variables on regression analysis (P ≤ .002 for all).

**Table 3:** Variables and their association with Neisseria gonorrhoeae in men dL: deciliter; ED: Emergency department; EMS: Emergency medical services; HPF: High-power field; mg: milligram; NA: Not applicable; OR: Odds ratio; PCP: Primary care physician; SD: Standard deviation; STD: Sexually transmitted disease; STI: Sexually transmitted infection; UTI: Urinary tract infection; vs: versus; WBC: White blood cell; y: year. Adjusting for age, race, and emergency severity index score. ORs for continuous variables are stated as per unit change in regressor.

	+ N gonorrhoeae	− N gonorrhoeae	P Value	Adjusted OR (95% CI)	Adjusted P Value
Age, mean (SD), y	26.4 (7.2) (n = 418)	31.5 (11.2) (n = 2,703)	< .001	.94 (.93-.96)	< .001
Black race (vs not Black)	93.0% (387/416)	86.1% (2,312/2,685)	< .001	1.90 (1.26-2.85)	.002
Married/life partner (vs not married)	4.6% (19,418)	9.3% (249/2,687)	.001	.96 (.58-1.59)	.86
Emergency severity index score, mean (SD), level 1-5	3.8 (.5) (n = 392)	3.6 (.6) (n = 2,558)	< .001	1.46 (1.21-1.77)	< .001
Having a (PCP) (vs none)	17.5% (73/418)	18.7% (505/2,703)	.59	.91 (.69-1.22)	.54
Arrived to ED by EMS/police (vs other)	1.5% (6/412)	3.8% (103/2,681)	.01	.46 (.18-1.17)	.10
ED triage pain scale, mean (SD) (0-10)	2.4 (3.5) (n = 27)	3.5 (3.8) (n = 231)	.17	.77 (.47-1.26)	.30
Mentioned “STD,” “STI,” “sexually transmitted,” “penis,” or “discharge” at ED point of contact (vs not)	65.6% (274/418)	44.4% (1,201/2,703)	< .001	1.77 (1.39-2.25)	< .001
Mentioned “UTI,” “bladder infection” or “pain,” “urinary tract infection,” “cystitis,” “dysuria,” “frequency” or “frequent urination,” or “urgency” at ED point of contact (vs not)	4.8% (20/418)	4.6% (125/2,703)	.90	1.59 (.95-2.66)	.07
Mentioned “abdominal pain,” “abdominal discomfort,” or “abdominal cramping” at ED point of contact (vs not)	4.1% (17/418)	6.0% (163/2,703)	.12	.93 (.55-1.58)	.80
Diagnosed in the ED with a urinary tract infection (vs not)	8.6% (36/418)	4.2% (113/2,703)	< .001	2.87 (1.88-4.39)	< .001
Treated with metronidazole in the ED or given it as an outpatient prescription (vs not)	44.5% (186/418)	28.9% (780/2,703)	< .001	1.63 (1.30-2.05)	< .001
Treated for gonorrhea and chlamydia (vs not)	90.7% (379/418)	57.6% (1,558/2,703)	< .001	6.40 (4.46-9.19)	< .001
Infected with N gonorrhoeae (vs not)	3.9% (5/127)	9.5% (63/662)	.04	.32 (.10-1.06)	.06
Infected with T vaginalis (vs not)	27.3% (114/418)	12.6% (341/2,701)	< .001	1.94 (1.49-2.51)	< .001
Urinalysis					
WBC count (cells/HPF), mean (SD)	72.3 (36.3) (n = 192)	23.2 (31.3) (n = 846)	< .001	1.03 (1.03-1.04)	< .001
WBC clumps present (vs not)	18.0% (34/189)	6.7% (56/839)	< .001	3.65 (2.16-6.16)	< .001
Mucus (0, 1+, 2+, 3+, or 4+), mean (SD)	1.2 (1.4) (n = 192)	1.2 (1.4) (n = 846)	.49	.87 (.77-.99)	.04
Leukocyte esterase (0, 1+, 2+, or 3+), mean (SD)	2.1 (1.1) (n = 204)	0.4 (.8) (n = 1,628)	< .001	3.99 (3.39-4.70)	< .001
Bacteria (0, 1+, 2+, 3+, or 4+), mean (SD)	0.6 (.8) (n = 192)	0.6 (.9) (n = 845)	.51	1.07 (.87-1.31)	.53
Nitrite positive (vs negative)	1.9% (4/208)	1.8% (30/1,641)	.79	2.86 (.92-8.89)	.07
Blood (0, 1+, 2+, or 3+), mean (SD)	.4 (.6) (n = 205)	.3 (.8) (n = 1,625)	.66	1.28 (1.04-1.56)	.02
Red blood cells (cells/HPF), mean (SD)	10.2 (17.5) (n = 189)	11.8 (25.1) (n = 846)	.30	1.00 (.99-1.01)	.91
Squamous epithelial cells (cells/HPF), mean (SD)	.1 (0.4) (n = 36)	1.3 (2.9) (n = 238)	< .001	.33 (.12-.91)	.03
pH (5-9), mean (SD)	6.1 (.9) (n = 208)	6.0 (.9) (n = 1,642)	.19	1.02 (.86-1.21)	.81
Bilirubin (0, 1+, 2+, or 3+), mean (SD)	.1 (.5) (n = 208)	.05 (.3) (n = 1,637)	.09	1.46 (1.02-2.08)	.04
Urobilinogen (0, 2, 4, 8, or 12 mg/dL), mean (SD)	1.2 (1.9) (n = 208)	.8 (1.5) (n = 1,641)	.002	1.09 (1.00-1.18)	.06
Protein (0, .5+, 1+, 2+, 3+), mean (SD)	.42 (.6) (n = 208)	.31 (.6) (n = 1,641)	.02	1.42 (1.13-1.79)	.003
Glucose present (vs not)	2.9% (6/208)	4.8% (78/1,639)	.29	.83 (.32-2.19)	.71
Ketones (0, .5+, 1+, 2+, 3+), mean (SD)	.1 (.3) (n = 208)	.1 (.4) (n = 1,639)	.16	.83 (.52-1.32)	.43

The urinalysis findings that were significantly different between those with and without *N gonorrhoeae* on univariable analysis were higher WBCs, more WBC clumps, higher leukocyte esterase, fewer squamous epithelial cells, and higher urobilinogen (P ≤ .002 for all). On regression analysis higher WBCs, less mucus, more WBC clumps, higher leukocyte esterase, higher blood, fewer squamous epithelial cells, higher bilirubin, and higher protein were significant (P ≤ .04 for all).

N gonorrhoeae and/or C trachomatis

Patients infected with *N gonorrhoeae* and/or *C trachomatis* were younger (25.9 vs 32.4 years), of Black race (91.7% vs 85.6%), less likely to be married (3.7% vs 10.2%), had higher ESI scores (3.8 vs 3.6), were less likely to come to the ED by EMS or police (1.7% vs 4.1), and were more likely to be diagnosed with a UTI (8.3% vs 3.7%) on univariable analysis than those uninfected with neither *N gonorrhoeae* nor *C trachomatis* (P ≤ .001 for all) (Table 4). Younger age, Black race, higher ESI, and diagnosed with a UTI were the significant variables on regression analysis (P ≤ .001 for all).

**Table 4:** Variables and their association with Neisseria gonorrhoeae or Chlamydia trachomatis, or both, in men dL: deciliter; ED: Emergency department; EMS: Emergency medical services; HPF: High-power field; mg: milligram; NA: Not applicable; OR: Odds ratio; PCP: Primary care physician; SD: Standard deviation; STD: Sexually transmitted disease; STI: Sexually transmitted infection; UTI: Urinary tract infection; vs: versus; WBC: White blood cell; y: year. Adjusting for age, race, and emergency severity index score. ORs for continuous variables are stated as per unit change in regressor.

	+ N gonorrhoeae and/or C trachomatis	− N gonorrhoeae and/or C trachomatis	P Value	Adjusted OR (95% CI)	Adjusted P Value
Age, mean (SD), y	25.9 (6.8) (n = 761)	32.4 (11.4) (n = 2,371)	< .001	.92 (.91-.94)	< .001
Black race (vs not Black)	91.7% (695/758)	85.6% (2,014/2,354)	< .001	1.63 (1.21-2.19)	.001
Married/life partner (vs not married)	3.7% (28/758)	10.2% (240/2,358)	< .001	.78 (.51-1.20)	.26
Emergency severity index score, mean (SD), level 1-5	3.8 (.5) (n = 719)	3.6 (.7) (n = 2,242)	< .001	1.36 (1.17-1.58)	< .001
Having PCP (vs none)	17.1% (130/761)	19.0% (451/2,371)	.24	.85 (.68-1.08)	.18
Arrived to ED by EMS/police (vs other)	1.7% (13/754)	4.1% (96/2,350)	.001	.53 (.28-1.01)	.05
ED triage pain scale (0-10), mean (SD)	2.4 (3.5) (n = 45)	3.6 (3.8) (n = 213)	.05	.92 (.73-1.15)	.45
Mentioned “STD,” “STI,” “sexually transmitted,” “penis,” or “discharge” at ED point of contact (vs not)	62.4% (475/761)	42.4% (1,005/2,371)	< .001	1.75 (1.45-2.12)	< .001
Mentioned “UTI,” “bladder infection” or “pain,” “urinary tract infection,” “cystitis,” “dysuria,” “frequency” or “frequent urination,” or “urgency” at ED point of contact (vs not)	4.6% (35/761)	4.6% (110/2,371)	> .99	1.36 (.88-2.09)	.17
Mentioned “abdominal pain,” “abdominal discomfort,” or “abdominal cramping” at ED point of contact (vs not)	4.1% (31/761)	6.3% (150/2,371)	.02	.82 (.54-1.25)	.36
Diagnosed in the ED with a urinary tract infection (vs not)	8.3% (63/761)	3.7% (87/2,371)	< .001	3.67 (2.49-5.41)	< .001
Treated with metronidazole in the ED or given it as an outpatient prescription (vs not)	39.9% (304/761)	28.1% (667/2,371)	< .001	1.45 (1.20-1.75)	< .001
Treated for gonorrhea and chlamydia (vs not)	84.6% (644/761)	54.9% (1,301/2,371)	< .001	4.03 (3.20-5.07)	< .001
Infected with T vaginalis (vs not)	5.7% (12/210)	9.7% (56/580)	.09	.60 (.28-1.29)	.19
Urinalysis					
WBC count (cells/HPF), mean (SD)	55.1 (38.8) (n = 344)	20.9 (31.1) (n = 700)	< .001	1.03 (1.02-1.03)	< .001
WBC clumps present (vs not)	11.8% (40/340)	7.4% (51/694)	.03	1.89 (1.13-3.17)	.02
Mucus (0, 1+, 2+, 3+, or 4+), mean (SD)	1.3 (1.4) (n = 344)	1.2 (1.3) (n = 700)	< .001	.94 (.85-1.05)	.28
Leukocyte esterase (0, 1+, 2+, or 3+), mean (SD)	1.5 (1.2) (n = 403)	.4 (0.8) (n = 1,437)	< .001	2.91 (2.56-3.32)	< .001
Bacteria (0, 1+, 2+, 3+, or 4+), mean (SD)	.5 (0.8) (n = 344)	.6 (0.9) (n = 699)	.06	1.00 (.84-1.19)	.99
Nitrite positive (vs negative)	1.0% (4/410)	2.1% (30/1,447)	.21	1.25 (.40-3.91)	.70
Blood (0, 1+, 2+, or 3+), mean (SD)	.3 (0.7) (n = 407)	.3 (0.8) (n = 1,431)	.90	1.25 (1.06-1.48)	.008
Red blood cells (cells/HPF), mean (SD)	10.0 (20.1) (n = 341)	12.4 (25.9) (n = 700)	.11	1.00 (.99-1.00)	.61
Squamous epithelial cells (cells/HPF), mean (SD)	.6 (2.7) (n = 85)	1.4 (2.6) (n = 192)	.03	.91 (.78-1.05)	.21
pH (5-9), mean (SD)	6.2 (.9) (n = 410)	6.0 (.9) (n = 1,448)	.007	1.07 (.94-1.21)	.33
Bilirubin (0, 1+, 2+, or 3+), mean (SD)	.09 (.4) (n = 410)	.05 (.3) (n = 1,443)	.04	1.42 (1.04-1.94)	.03
Urobilinogen (0, 2, 4, 8, or 12 mg/dL), mean (SD)	1.1 (1.7) (n = 410)	.8 (1.5) (n = 1,447)	< .001	1.07 (.99-1.14)	.07
Protein (0, .5+, 1+, 2+, 3+), mean (SD)	.4 (.6) (n = 410)	.3 (.6) (n = 1,447)	.02	1.34 (1.11-1.62)	.002
Glucose present (vs not)	1.5% (6/410)	5.4% (78/1,445)	< .001	.45 (.18-1.17)	.10
Ketones (0, .5+, 1+, 2+, 3+), mean (SD)	.1 (.3) (n = 410)	.1 (.4) (n = 1,445)	.34	.91 (.66-1.27)	.59

The urinalysis findings that were significantly different between those with *N gonorrhoeae* and/or *C trachomatis*, compared with those without *N gonorrhoeae* and *C trachomatis* were WBCs, more frequent WBC clumps, more mucus, higher leukocyte esterase level, fewer squamous epithelial cells, higher urine pH, higher bilirubin, higher urobilinogen, higher protein, and less likely to have glucose (P ≤ .04). On regression analysis higher WBCs, more WBC clumps, higher leukocyte esterase, higher blood, higher bilirubin, and more protein were significant (P ≤ .03 for all).

Significantly more patients infected with *N gonorrhoeae* and/or *C trachomatis* were treated with antibiotics for the infection in the ED (84.6% vs 54.9%) than those who had neither infection (P < .001). Infection with *N gonorrhoeae* and/or *C trachomatis* was not significantly associated with infection with *T vaginalis*.

## Discussion

In this study, 24.3% of tested men were infected with either *N gonorrhoeae* and/or *C trachomatis*, and 3.6% were coinfected with *N gonorrhoeae* and *C trachomatis*. There were significant differences in the demographics, patient characteristics, and laboratory findings of men with and without *N gonorrhoeae* and *C trachomatis*. Younger age, Black race, and unmarried status were all significant risk factors for infection with either *N gonorrhoeae* and *C trachomatis* on univariable analysis. Our results are supported by previous research showing that the highest STI infection rates in men were for those aged 20 to 24 years [1]. Being of Black race was a significant risk for *N gonorrhoeae* and *N gonorrhoeae* and/or *C trachomatis*, but not for *C trachomatis* alone on regression analysis, although race approached significance (P = .06). Black men were previously found to be 8.5 and 6.8 times more likely to have *N gonorrhoeae* and *C trachomatis* than White men [1]. In our study, having a primary care physician (PCP) was not associated with *N gonorrhoeae* and *C trachomatis*, which contrasts with other reports showing that women unable to name their PCP were at increased risk for STIs [17].

Patients infected with *N gonorrhoeae* but not with *C trachomatis* were associated with *T vaginalis* co-infection than patients without those infections. Little is known about the epidemiology of *T vaginalis* in men in the ED [7,10].

On regression analysis, patients with *C trachomatis* and *N gonorrhoeae* had a 2.67 and 6.40 odds, respectively for being treated for their respective infections in the ED. This may reflect the higher rates of symptomatic infection in men with *N gonorrhoeae* than men with *C trachomatis* [18]. The majority of men (84.6%) infected with *C trachomatis *and/or *N gonorrhoeae* were appropriately treated for the infection in the ED, but 54.9% were treated and did not have an infection.

There are no externally validated clinical decision rules for men who should undergo testing for *N gonorrhoeae* and *C trachomatis* in the ED. Although over a dozen STI prediction models exist, two were developed using ED patients [19,20]. One model used a point system for education, age, marital status, nonantibiotic use in the past month, non-ED as a primary source for health care, new sexual partner within the past two years, dysuria, or discharge in the past three months [21]. The other model was specifically for *N gonorrhoeae* and *C trachomatis* in men in the ED and used the following variables: ≤24 years of age, penile discharge, lack of health insurance status, and contact with someone infected with *C trachomatis* or *N gonorrhoeae* [20].

We identified an association between urine leukocyte esterase and infection with *N gonorrhoeae* and *C trachomatis* on univariable and multivariable regression analyses, and this has previously been suggested to be a screening tool for gonorrhea and chlamydia in asymptomatic men but when used in isolation lacks sufficient sensitivity and specificity [15]. The urine WBC count was associated with *N gonorrhoeae* and *C trachomatis* on univariable analysis, but only with *N gonorrhoeae* on regression analysis. It is possible that these results are related to a stronger inflammatory response caused by *N gonorrhoeae* compared with *C trachomatis* in most patients [13,18,22].

Limitations

All data were retrospective and collected from northeast Ohio. Only data available in the hospital medical record were available for analysis so information on detailed sexual health were unavailable. The dataset also lacked racial diversity. Not all patients were screened for *T vaginalis*, so the prevalence of coinfection with *T vaginalis* and *N gonorrhoeae* and *C trachomatis* could not be determined. The sensitivity and specificity of the Aptima Combo2 assay is about 96% to 99% sensitive and specific so there are likely some false-positive and negative results in the dataset [13]. Patient history and physical examination findings were not included in the dataset, and patients empirically treated for STIs in the ED without testing were not included.

## Conclusions

In the absence of point of care testing clinicians need to decide whether to empirically treat for STIs or wait for the test results. Age, race, being diagnosed with a UTI, and ESI scores plus higher WBCs, higher leukocyte esterase, higher bilirubin, and higher protein on the urinalysis may be helpful when estimating risk for *N gonorrhoeae* and/or *C trachomatis* infection in men in the ED. While most men with *N gonorrhoeae* and *C trachomatis* were correctly treated with antibiotics in the ED, a majority of patients without *N gonorrhoeae* and *C trachomatis* were also inappropriately given antibiotics.
